# Being Well, Looking Ill: Childbirth and the Return to Health in Seventeenth-century England

**DOI:** 10.1093/shm/hkw086

**Published:** 2017-01-15

**Authors:** Leah Astbury

**Keywords:** childbirth, early modern, health, recovery, life-writing

## Abstract

For a month after childbirth, the authors of medical and religious prescriptive literature instructed new mothers to keep to their beds. During this time they were expected to bleed away the bodily remnants of pregnancy. At the end of this month writers considered women ‘well’. Bleeding, in this definition, was commensurate with recovery. This article shows that although in prescriptive material, maternal health was measured according to this process of purging, for early modern middling and upper sort women and their families, the bodily effects of childbearing continued to impede their ability to return to normal household tasks and behaviours long after the ritual month of ‘lying-in’ had ended. Using life-writing, casebooks and vernacular medical literature, this article challenges prevailing notions of what it meant to recover in early modern England, arguing that women’s ‘childing’ or ‘childebed’ narratives only ended when they perceived their bodies to be unaffected by pregnancy and labour.

In July 1647, Dr Denton, physician to the aristocratic Buckinghamshire Verney family, wrote to Ralph Verney relaying that Ralph’s wife, Mary, who had given birth the month before was ‘churcht [churched] & well, but lookes ill enough’.^1^ The month after childbirth in early modern England has been represented by historians as one of complex social inversion, a time of feminine collusion in which the newly delivered mother was sheltered from her normal social and household engagements.^2^ In medical terms, this month, termed ‘lying-in’, was the period time in which women ought to be confined to bed and bleed away the remnants of pregnancy. Mary was ‘churched’ meaning she had undergone the religious ceremony held at the end of lying-in, in which family and friends accompanied the new mother as she left the house to journey to church to thank God for her safe deliverance. This ceremony, interchangeably called ‘churching’ or ‘thanksgiving’ in the seventeenth century—‘purification’ prior to the 1552 *Booke of Common Prayer—*supposedly marked the moment when a mother returned to her normal household activities. There were few financial constraints on an aristocratic woman like Mary, but for poorer women, as Laura Gowing has noted, ‘lying-in for a month was probably impracticable’.^3^ Work or other obligations would almost certainly have curtailed the period of recuperation and necessitated quieter and less extravagant celebrations.

Denton’s observations reveal three separate issues in early modern understandings of recovery from childbirth—being ‘well’, feeling and behaving healthily, and finally, the religious and social ritual of churching. Sara Read, drawing from literature, printed vernacular medical treatises and doctors’ casebooks, has convincingly argued that the term ‘well’ was a euphemistic way to convey that Mary had finished bleeding.^4^ For Read, Denton’s confluence of churching with the cessation of bleeding serves to support her argument that important moments of social and personal transition for early modern women were marked by bleeding. I would suggest these observations reveal that despite the dominance of the prescriptive framework of a month to recover, for many women the cessation of postpartum bleeding and churching did not correspond with when they looked or felt well.

Despite the wealth of material on pregnancy and the social rituals of childbirth, the bodily and emotional experience of the period after birth has been relatively neglected. The cultural meaning of the ceremony of churching, for example, has garnered significant attention from historians. Keith Thomas argues that although the ceremony was officially re-labelled thanksgiving, for the laity it was ‘indubitably a ritual of purification’.^5^ Patricia Crawford has suggested women merely submitted to the practice despite fundamental religious changes brought about by the Reformation, while her earlier work argued that menstruating bodies provoked distrust and disgust from men.^6^ Churching stopped in 1645 and was reinstated with the Restoration, at which point, David Cressy argues, the ceremony became more about ‘conformity to ecclesiastical discipline’ than the potential spiritual benefits of the practice. For Cressy, churching held intellectual power for women, not as a way to remedy impurity, but because it gave them a moment in the spotlight. He shows that a great many families continued churching ceremonies in the turbulent period between 1645 and 1660—Mary Verney for one.^7^ Indeed, both Cressy and Adrian Wilson represent churching as celebratory rather than regulatory and a ceremony that persisted because ‘women wanted it for religious, cultural and emotional reasons of their own’.^8^

These accounts have done much to elucidate how official ceremonies interacted with the beliefs and practices of the laity. But they assume without qualification that a month after birth, women were, and perceived themselves, to be well again. Cressy notes, for example, that churching ‘involved the church and the community in her recovery’.^9^ Similarly, Gail Kern Paster explains that the ceremony ‘continued symbolically to mark—if not explicitly to signify—a moment of bodily restoration, the cessation of flow, a social return (however temporary) to a nonpregnant state’.^10^ She states that the purification element of the ceremony was explicitly about the end of the ‘postpartum flow’ but formally marked the end of a childbearing experience.^11^ This neglects to consider the ways in which childbearing was a fluid, ongoing process and that women’s bodies could continue to be effected by birthing long after their formal confinement was up.

Examining the correspondence, diaries, journals and spiritual meditations of women and their families, often termed ‘life-writing’, suggests that recovery and restoration was a far more complex process. Women perceived their bodies to be in childbed until they had returned to their former selves, which was not always simplistically defined according to whether they had bled sufficiently. This might occur before the month was up, or years after bearing a child.

The seventeenth century witnessed many important changes in personal writing practices, including a ‘significant extension of letter-writing skills throughout society’ and an ‘expansion in the range of uses to which letters were put’, a development which has been tied to certain Protestant traditions of self-examination and evaluation.^12^ Such texts often combined observations of daily life with soul-searching investigation of personal sin. Writing about one’s own or others’ health was common in correspondence and in diaries, which has made life-writing a useful source for the social history of medicine.^13^ Childbirth was part of this dialogue, and it is important to note that both women *and* men wrote about childbearing in correspondence, journals and diaries. Rather than being problematic, this article shows that male interest and discussion of parturient and recovering bodies reveals the family was a far more important context for having babies than the female communities which have dominated previous histories of birthing. What I term ‘childbearing narratives’ were interwoven in family affairs and reputation in complex ways in life-writing. I use the term ‘family’ capaciously, in the same way as early modern individuals used it, to mean not just blood relatives, but those who were within the emotional and practical boundaries of the household. As Naomi Tadmor states the ‘concept of the [early modern] family emanated from relationships of co-residence and authority’, often including servants.^14^

Such sources are not without their difficulties. It is important to remember, as Adam Smyth has shown, how accounts of life events might constantly be revised and sit ‘within a web of other life-writing forms’.^15^ Similarly, authors might write diaries and journals with the view they would be read by family and/or community members. Alice Thornton, for example, a seventeenth-century Yorkshire gentlewoman, explicitly wrote her meditations to disprove damaging rumours which besmirched her honour.^16^ Because of this, some historians have argued that life-writing reveals less about an individual or family’s personal reception of an event, such as childbirth, and more about cultural scripts.^17^ Read carefully, however, these texts are fruitful sources for the interaction between the emotions and health.

Another difficulty of using life-writing sources is that they originate from a particular elite sector of society that valued written self-examination and accounting. It is difficult to uncover the experiences of less wealthy families. In this way, it is crucial not to universalise about experience in early modern England; this is primarily a story about middling and upper sort families. Considering the literate elite, however, provides an opportunity to consider the ways in which medical and religious prescription and practice interacted. In addition to life-writing, this article makes use of vernacular midwifery manuals printed in England, a genre that would not have been out of the financial means of these families. The publication of medical texts increased markedly in the late sixteenth and seventeenth centuries. Mary E. Fissell has estimated that by 1700 there was one vernacular medical work in circulation in England for every four families.^18^ Of particular currency were the guides directed to midwives but also women in ‘child-bed’, attempting to explain generation.

This article argues that although the prescriptive framework of a month was dominant in the ways in which women, their families and practitioners understood postpartum bleeding, it did not necessarily correlate with their recovery. Examining the life-writing of seventeenth-century men and women reveals that bodies ‘looked’ or felt disordered, weak and sickly long after birth. Hannah Newton has recently called into question the historical neglect of recovery from disease in contrast to the wealth on illness, diagnosis and cure.^19^ One reason for this must undoubtedly be that although indviduals could be free from disease and therefore be in a Galenic ‘neutral’ or ‘healthy’ state, their ability to function might continue to be impaired indefinitely. This article complicates our understanding of what it meant to be healthy in early modern England.

The first section of this article explores the prescriptions in childbearing manuals for how women ought to be ‘ordered’ immediately after birth. I argue that divesting the maternal body of the remnants of pregnancy steadily and without retention was paramount to restoring health. The second section looks at circumstances in which this bleeding was considered unhealthy: when it exceeded the month long lying-in period. By bringing entries in doctors’ casebooks to bear on life-writing, I show how women and physicians took action to stem bleeding a month after birth. I discuss the terminology women used to describe the period in which they were affected by childbearing—Alice Thornton described the period of more than 20 weeks in which she struggled to regain health her ‘childebed’.^20^ The final section looks at the lingering ailments which could trouble women and how the health of other family members had a bearing on the rate at which women understood their bodies to be recovering.

## Prescriptive Understandings of Maternal Health

Delivery did not end with the birth of a child. It continued through the excretion of the after-birth and blood that had nourished the infant in the womb called lochia. ‘Presently after she is delivered, labor to make the After birth follow’, Nicholas Culpeper the famous herbalist instructed.^21^ The prompt extraction of the placenta or secundine was not simply desirable; it was paramount. If a midwife or physician failed in this task, the life of the woman was ‘much indangered’.^22^ The midwife Jane Sharp in her 1671 treatise warned other midwives that they ought not to be complacent after childbirth for ‘Women are as in as great danger if not more, after the young is born’ than during delivery.^23^ If a midwife cut the navel cord of the infant too hastily after birth without holding the maternal end in her hand, it would be ‘drawn back into the Womb, and hid there with the Secundine’.^24^

The placenta was described as highly sensitive and liable to refuse to exit the womb at the slightest provocation by mother or midwife; in a sense it had agency. John Pechey, a Sussex physician, presented the reader in 1696 with a long list of triggers that would cause a placenta to misbehave: if the caul (umbilical sac) of the infant was too thick; if the labour was particularly hard; if the mother experienced ‘violent passions of the Mind’; if she had poor birthing posture; if the child was particularly large and ‘lusty’; if the woman was subjected to ‘Coldness of Air’; or if perfumes were administered ‘that allured the womb upwards’ before full excretion had occurred.^25^

If one of these complications occurred and a woman was improperly purged, childbearing manuals described the complete bodily disorder and disruption that would ensue. Sleep would be fitful or non-existent. Culpeper explained that the flux stirred the humours and led to emotional distress and restlessness. The mother might also be gripped by fear and anxiety.^26^ Vomiting after birth was noted to be particularly common. Newly delivered women often ‘cast up crude and indigested meat’, caused by the ‘weakness of the stomach by the womb’ and sometimes from the ‘humors that come to the stomach, from parts near the womb’. This was thought to occur when ‘the after flux doth not flow’ and the blood that might be excreted in the form of lochia ‘goes to the great veins and liver’ which in turn might flow into the stomach’.^27^ Laxatives were used to encourage the process of purging. Sharp warned that while ‘senetives’ were helpful after birth, if they were too aggressive this could cause a sudden catastrophic loss of blood, called ‘flooding’.^28^

If a woman could not be provoked to bleed, vomit or was costive, Culpeper recommended ‘friction of the legs, ligatures and Cupping with Scarification’ in the aim of stirring up the bad humours through agitation.^29^ Expelling humours in this way amounted to vicarious or deviated menstruation. In Cathy McClive’s terms, blood ‘sought an alternative exit from the body if the usual genital pathway was blocked’.^30^ Newly delivered women were not just still in their ‘childebed’ or ‘travail’ in the hours and days after a baby had been born, but they were also perceived to be delivering while the placenta and lochia were within them. Sharp explained how mothers would experience waves or pangs of pain, similar to giving birth, as the womb expelled the placenta. These pangs, she explained, came from the vessels and membranes ‘by which the womb hangs’.^31^ If there was ‘clotted blood detained’, ‘sharp blood sticking to the womb’ or cold air trapped within, the pain would be forceful and hard.^32^ These pains were necessary; indeed, they were encouraged by purges, laxatives and scarification.

The contents of the womb were described as menstrual blood that was ‘filled daily more and more till the Birth’ with the infant’s sweat and urine.^33^ The loss of this ‘foul liquor’, vaginally, was called ‘lochia’.^34^ Ideally, its flow would follow the expulsion of the placenta and would continue steadily for a month. The language that medical authors used to describe this blood loss was indistinguishable from the lexicon of menstruation: it was her ‘terms’, ‘flowers’, ‘courses’ or ‘the flux’. The shedding of the placenta and this blood were both part of processes of care aimed at ‘governing’ or stabilising newly delivered women. To retain the lochia, as with the placenta, was deemed unequivocally fatal. Pechey described how the ‘suppression of the lochia is one of the worse Symptoms that can befall a Woman in Child-bed’.^35^ If the flow of blood ceased after three or four days, the time ‘they should come down plentifully’, the consequences for maternal health were grave. Just as with the retention of the placenta, remaining lochia, Pechey stated, caused a ‘putrid fever’, nausea, fainting spells, shaking, frenzied behaviour, a desire to ‘eject something’ along with a ‘sense of heat and pain’.^36^ Finally, the corrupting matter within her would emit a ‘cadaverous smell’. The ‘Coldness of the extream parts’ was the last sign before convulsive fits and death.^37^

Postpartum bleeding was crucial to survival after childbirth for two reasons. One was obvious: pregnancy had deprived women of their usual monthly periods, meaning there was a backlog to expel. When not pregnant, monthly evacuations removed harmful humours. Indeed, Gianna Pomata has suggested that menstruation gave women an advantage in hastening the Hippocratic-Galenic moment of ‘crisis’—a turning point in a disease, which might make recovery from illness more speedy, something reiterated by Wendy D. Churchill.^38^ Secondly, and more importantly, these missed periods were concocted and consumed by the infant for nourishment, before it added its own sweat and urine into the mix. The bodily matter of pregnancy was no longer part of the maternal body; it was alien to it, and corrupting.

Crucially, not all postpartum blood loss was perceived as healthy. In contrast to lochial bleeding that was beneficial because it removed corrupted matter, flooding was seen as life-threatening because a mother would lose her own blood on top of the products of excretion. Pechey warned that, ‘Flooding is a more dangerous accident than any other which may happen to a woman newly laid’. He explained that flooding was ‘when nothing remains behind in the Womb, the Blood notwithstanding continues to flow’. Pechey made explicit the link between the health benefits of purging—removing the matter remaining in the womb—and unhealthy purging—emptying the womb and then depleting the new mother. The only cure in his understanding was bloodletting, which would cool the humours, and draw blood away from the womb.^39^

In medical texts, a significant proportion of discussion of maternal health after birth was devoted to outlining stipulations for how long lochial bleeding should continue. Authors interchangeably used scriptural and ancient sources. Jane Sharp gestured to Leviticus 12:1–5 which proposed 33 days of blood loss after the birth of a boy and 66 days if it was a girl; but she simultaneously referenced Hippocrates who suggested 30 days for a male child and 42 days for a female.^40^ This was because female infants were understood to be naturally wetter and thus they produced more fluid in the womb, while male infants were substantially hotter and drier. To further complicate recommendations, many contemporary writers suggested that women that breastfed would bleed for a shorter period of time. Breast milk was understood to be the same menstrual blood that had nourished the child in the womb, concocted and purified in the breasts, and therefore breastfeeding diminished the amount of blood that needed to be purged.^41^ These stipulations often sat side by side without resolution in medical texts.

The construction of a month to bleed and recover appears monolithic in medical texts, and yet, even within this narrative there was room to manouevre; for recovery that was fast or sluggish to be perceived as unalarming, and for women to read their own bodies and assess whether they had truly returned to health. Culpeper thus followed his prescriptions with the qualification that ‘this is not alike in all, differs in respect of age and diet’.^42^ Sharp similarly prefaced a chapter on ‘How women after Child-birth must be governed’ with the admission that ‘There is great differences in Womens constitutions and education; you may kill one with that which will preserve the other.’^43^

## Rates of Recovery

The ‘differences between women’, in Sharp’s terms, meant some bodies were thought to return to their former selves more quickly than others after birth. This was borne out in life-writing. To say that a woman had been ‘safely delivered’ was a common phrase in family correspondence. It referred not just to the act of surviving delivery, but was an assessment that the woman was displaying promising signs of restoration. Thus, when Charles Cheyne relayed his wife’s delivery to his brother-in-law in 1656, he noted that she was ‘well and safely brought a bed of a daughter’.^44^ Similarly, when Charles Hatton wrote to his brother, Christopher, about the birth of a daughter, Christopher’s wife was remarked to have been ‘safely delivered’.^45^ In these letters, being ‘safely delivered’ did not denote that a mother was free from discomfort or pain, although, midwives or nurses could be sent away earlier than they might normally. When Thomas Smyth wrote to Edward Phelips in 1641, for example, he relayed that his wife, Florence, was faring so well after giving birth that they planned to ‘dispatch away her midwife’ within the week. He hoped that this news would ‘settle my cousens mynde’.^46^ Surviving birth, however, only marked the beginning of a recovery process. Francis Thornhough of the aristocratic Bedfordshire St John family wrote to his brother-in-law Oliver St John congratulating him on the birth of a daughter. He was relieved that his sister had had a ‘safe delivery’. Thornhough hoped that ‘God will still continue good unto her in the restoring her to her former strength’.^47^ In this way, even after the child and placenta had been delivered and lochial bleeding commensed, the struggle to regain health was not over.

In many letters, families constructed larger childbearing narratives by referring to previous births and recoveries. Mary Hatton, for example, wrote to her brother Christopher in 1676 after her sister’s birth, stating that ‘my sister comes so quick […] you may have great reason to hope that within a year to have a son’. Likewise, Thomas Smyth thanked God in a letter to his brother Edward for his wife’s ‘easy partinge with her fruit when tis ripe’, and noted that she had recovered so quickly that he would soon be ‘trying my skill for another boy’.^48^ Florence’s precocious recovery allowed him to try his ‘skill’, or resume marital relations, quicker than he might have done otherwise. Her ‘easy partinge’ facilitated the performance of his own reproductive masculinity. Medical texts commonly argued that the more enjoyable sexual union was, the more likely it was to result in conception.^49^ In this way, Thomas was boasting that the couple enjoyed regular and passionate sex, which facilitated the speedy cycles of conception, birth and recovery.

This was undoubtedly a joke between friends, but the humour reveals certain important nuances and assumptions about the ideal birthing body in elite families in England in the seventeenth century. The joke that Florence returned to her former self so swiftly after labour may simply have been funny because Thomas was making a rather bald and crude allusion to sex. Other correspondence of the Smyth family reveals that by 1641 it had become a running joke that Florence and Thomas were doomed to only ever produce girls. When Thomas Smith, another male relative, wrote to Smyth in 1637 he jested ‘if you hold one (‘sic’), and my wife too, you’ll be able to people a whole country out of your daughters’.^50^ Such jibes reveal that what was humorous was not just that the pair were producing girls, nor that because Florence was well again they could have sex, but that there was something potentially embarrassing about ‘partinge’ with ‘fruit’ so easily.

An unobstructed birth and quick recovery demanded a womb that was yielding—it expelled a baby, the placenta and lochial blood steadily and without retention. A birth that was too swift and painless could, however, be shameful. Laura Gowing in her work on the cultural recognition of pregnancy has suggested that unmarried women accused of infanticide claimed to have had ‘short, painless or unexpected labour’ both to explain why they had not called for assistance, but also to support the argument the child had been born dead.^51^ She adds that,

It was also established knowledge that poor women, and in particular mothers of bastards, bore their children quickly and more easily than those fully prepared for a lying-in: stories of illegitimate births and the murder of new-borns created a culture in which such labours were meant to be shamefully easy.^52^

For these women their babies might ‘slip’ from them; their wombs were slack and open in a way that was problematic and troubling. At the other end of the spectrum, Thomas Bentley’s 1582 *Monument of Matrones*, a book of prayers for breeding women, described labour as slow, torturous and painful, but fundamentally spiritually redemptive.^53^ Thomas’ description of Florence’s womb is a rather graphic description of a childbearing experience that was somewhere in between the furtive, slippery births of those accused of infanticide and the slow, excruciating labours described in religiously prescriptive material. Florence’s womb parted with ease, but Thomas additionally suggested that this same characteristic openness also enabled enjoyable love-making. In another context this might have been shameful, but within this familial male exchange, it was intended as comedic. The rate of recovery of a newly delivered woman, and the speed at which she might conceive again, reflected maternal *and* paternal good health and the frequency and pleasure of their sex, but it also had broader implications for the reputation of the family as a whole.

When women failed to bleed after birth, life-writing shows that authors shared the fears of medical authors. Two weeks after the birth of their son, William, in July 1672, the Anglican minister Isaac Archer recorded that his wife, Anne, was ‘grievously sick, and faint’. Her illness was attributed to ‘some noxious and venomous impurities that nature should have cleansed her of’.^54^ Remnants of Anne’s pregnancy (either the placenta or lochial blood) made her unwell. So grave was her affliction, Isaac recorded, that her parents were sent for and the family prepared for her death. Domestic remedies were used, and a doctor called, but before he arrived ‘by degrees her fitts went away, and nature did it’s (‘sic’) office, without any other physick, except herbs boiled for such an use’.^55^

The example of Anne Archer reveals that although there was an understood variability in the speed of recuperation indicated in medical literature, and embedded in the childbearing narratives families constructed in life-writing sources, the need to bleed was seen as essential to surviving childbirth. As the term ‘safely delivered’ indicates, this was only the first stage in restoration: surviving was one thing, regaining health another. This meant that women who failed to bleed in the month after birth often sought the assistance of physicians.

## Bleeding for Too Long

The casebooks of John Hall are particularly valuable in examining medical expectations of recovery from childbirth.^56^ Hall recorded the details of 155 patients he attended to in Stratford-upon-Avon. They were of varying social backgrounds, from aristocracy to those of much humbler origins. Nearly two-thirds of the patients recorded in his casebooks were female, and many of these cases were related to recovery from childbirth.^57^ Physicians’ notes rarely recorded the spoken word of patients. However, as Olivia Weisser has observed, they reveal a process in which the practitioner ‘developed an explanation of the problem and how to treat it’. Weisser states, ‘This process was informed by the assumptions and observations both patient and healer brought to the interaction’, and therefore reveals an important moment in the construction and interpretation of narratives about childbearing.^58^

Women often sought Hall’s assistance when they failed to bleed after birth, or judged their lochia to be too light. For instance, Anne Jackson consulted Hall in the 1650s because she was ‘not well purged after birth’. She had suddenly fallen ‘into a grievous Delirium, no other disease preceding’. Hall recorded that ‘she was most angry with those that formerly she most love’ and ‘by intervals there was a Fever acut, which made me fear a Frenzy’. He prescribed a series of purges which had ‘happy success’.^59^ In a similar case, Cecily Hopper consulted Hall when her ‘After-birth was retained and corrupted, so that it was cast forth in little stinking bits, whence a direful stench ascended into her Stomach, Heart, Liver, Diaphragm.’ Hall noted that the corrupted humours had reached her brain, causing ‘Pain of the Head, often fainting, and cold sweats; so that there was danger of death.’^60^ Likewise, Mrs Chandler consulted Hall five days after her delivery with ‘Erratick Labour, with horror, heat, and shaking often day and night’.^61^ These symptoms are undeniably similar to those that medical authors suggested would occur when the placenta and lochia were retained (fever, nausea and pains reminiscent of delivery). The women who consulted Hall were ‘recovered’ in his words, through purges which encouraged the body to expel remnant pieces of afterbirth, blood and corrupted humours that had become resident as a result. The nature of casebooks means it is difficult to know whether women felt themselves ‘cured’ of the lasting bodily impacts of pregnancy and childbirth. Hall always represented himself as the end point in childbearing narratives in his casebooks: patients were either completely cured or died.

Fluids and humours and their attribution to infant or mother as medical agents, were at the centre of these assertions about whether a body was healthy or unhealthy. Implicit in these narratives was returning to one’s former self. By losing the bodily matter which had belonged to the infant, with its capacity to corrupt and pollute, a woman’s body was becoming autonomous. Shortly after the birth and death of another child in 1679, the previously discussed minister, Isaac Archer relayed that his wife ‘fell sick of a feaver and ague’. She was ‘taken senseless in the fit, and had cold clammy sweats, oppressions that stopped her’ and acted strangely. For ten days Anne languished and, again, the family prepared for her death, before miraculously, Isaac relayed, ‘she came to her selfe’.^62^

This example shows that although Anne was bodily ‘her selfe’ she might continue to look and feel unwell long after she had excreted remnants of pregnancy and corrupt humours. Anne was still weak, possibly even bedridden, and her struggle with poor health was not over. This is not to say that practice was completely at odds with prescription. Rather, that the concept of recovery embedded in seventeenth-century vernacular medical texts was primarily about humoural recuperation. The expectation that a newly delivered woman would return to her former self within a month of birth only referred to complete excretion of corrupting matter. It did not take into account other childbirth ills such as weakness, limping, breast ailments and tearing. These were not considered life threatening in medically prescriptive material, but continued to curtail the return to a former self.

These childbearing narratives often competed with the normative constructs implicit in printed medical literature, casebooks like Hall’s but also dominant religious frameworks which underpinned the ceremony of churching. It is in these moments, in which bleeding continued long after this month, that women sought the assistance of medical practitioners, suggesting an awareness that their bodies did not live up to prescriptive expectations. Mary Barnes, for example, consulted Hall, ‘being troubled with the over-flowing of her Courses’, a month after her delivery.^63^ Elizabeth Randolph troubled with ‘too much Flux of her Courses’, after giving birth, along with ‘Wind in the Stomach’.^64^ She was prescribed alum in red wine, an astringent applied internally and externally to stop the flux. In one case, Hall was explicit that Lady Sandys consulted him ‘after her Purification’ or churching ceremony, yet she continued to bleed profusely through haemorrhoids.^65^

Letters and diaries suggest a similar trend in seeking advice for excessive lochial bleeding a month after birth. Alice Thornton recorded in her manuscript meditations the painful ‘hemrides’ (haemorrhoids) which ‘daily lost about four or five ounces of blood’ which she was plagued with for more than 20 weeks after giving birth to her fifth child. On the instruction of her physician, Alice travelled to Scarborough to take the waters in the hope this would go some way in remedying ‘the excessive losse of blood and spirits, in childebed’.^66^ Thornton’s ailment was clearly different from the frantically disordered condition of the women who had not bled sufficiently. They were feverish, frenzied and acted strangely. Thornton, however, described herself as diminished, weak, unable to walk or support herself. Thornton’s doctor warned that her condition was potentially fatal and could render her infertile. The excessive bleeding after birth challenged her mobility and made her weak, however, it also had the potential to deprive Thornton of the heat and moisture within her womb needed for successive conceptions. Thornton termed this long, difficult struggle back to health her ‘childebed’. This is key to thinking about how early modern women constructed their childbearing experiences and understood their bodies in relation to dominant medical and religious discourses. She did not use the term lying-in; her formal month and churching had passed, suggesting this term was only used to refer to this period of time, and not as a synonym for recovery. For Alice these harmful corrupting forces had been excreted, but her childbearing narrative and journey to recovery continued.

In contrast to the ink spent on stressing the importance, time, consistency and benefits of lochial bleeding, medical writers were significantly more muted on what women could expect after they were perceived to be free of the remnants and blockages of pregnancy. A second stage of after birth care is implicit in childbearing guides: that of soothing and nourishing the body, something that could only be attempted once bleeding had been established. Whereas bleeding promised to save women from death, transform them and make them healthy, the poultices, baths and plasters devoted to healing ‘external’ ailments, like Thornton’s weakness, seem comparatively meek.

Tearing, prolapses and fistulae were represented by medical writers as troubling, but not life threatening. In this sense, ‘Looseness of the womb’ might lead to incontinence, but it was an external injury, and would not corrupt humours, or lead to disease.^67^ These tears and strains were simply inevitable ‘when the mother is tender, and the child is great’ and were only mended when bleeding was steady or she had been completely purged.^68^ Culpeper suggested that if a tear occurred during delivery, the mother should lie on her back ‘with her feet drawn up, with Sweets to her nose, and stinks to the womb, so the womb will be retained, and the flux continued’.^69^ Other treatments for external injuries were the application of astringents to tighten the maternal body, or ‘close the womb’ in Sharp’s terms, and ‘baths’.^70^ This practice of dividing after birth care into two phases can be observed strikingly in recommendations for baths for newly delivered women. Pechey provided instructions for a bath consisting of honey, roses and chervil suitable daily in the first eight days after birth.^71^ After the first eight days had passed and bleeding had been established, he proposed a regime of water and wine, applying a plaster to the belly and privies thereafter.^72^ The wine functioned to both heat the body and as an astringent, whereas the plaster encouraged the belly to return to its former shape. Such external ailments were represented to the reader as minor, a nuisance but not debilitating. Bleeding and removing the humours associated with pregnancy and birth was the main concern for medical practitioners: it was what really constituted prescriptive definitions of recovery. External injuries were more about ‘looking ill’, rather than being unwell.

## Prolonged Recoveries

Prolonged recoveries, whether brought about by insufficient purging or lasting weaknesses and ailments, caused significant disruption to family life. When James Yonge’s wife gave birth in 1681, he noted ‘this year my wife delivered of a daughter, she had a sickly and tedious lying-in by taking some cold’.^73^ The child was also unwell it seems, for he added that it was christened at home, shortly after birth. Home baptisms, especially shortly after birth, were usually only performed when it was judged improbable that the infant would survive. The disruption to normal and expected routine, in this case having to baptise the infant at home rather than at church, mirrored and reflected the internal bodily disorder of mother and child.

Other fathers noted that their daily duties and activities were curtailed by difficult and arduous recoveries. Charles Trelawny apologised to a male correspondent in November 1700 for ‘soe long neglecting; to owne the favour of your too (‘sic’) letters, but when I tell you, that the indisposition of my other Side, was the cause, I am sure you will forgiue me’. He explained, ‘My wife hath been very much out of order, euersince she was brought to bed, & the child hath been euen at death & door more than once since she was borne, and is at this minute upon the Rack w[i]th convulsive fits’.^74^ The infant’s illness he stressed ‘makes the mother almost mad’. The assertion that his wife was ‘very much out of order’ even for a woman who had recently given birth is striking. It yet again reveals the importance of notions of bodily order in definitions of health, but it also shows how individuals understood maternal well-being after childbirth as something that was measured according to an individual’s bodily norms, rather than solely by prescriptive ideals.

The theme of disruption to social arrangements was also prominent in female correspondence. When Elizabeth Smyth wrote her brother Thomas Smyth after their sister had suffered a late term miscarriage, Elizabeth noted that ‘she was made unable so soune to the travaille (travel)’ because ‘she was driven to laye in her bed a weeke after’.^75^ It is important that bleeding was not prominent in this explanation of poor health, but rather her feeling weak. Remaining still and indoors was seen as crucial to allow a woman’s own body to muster up its strength to expel any remnant humours.

This immobility, however, could prevent women from fulfilling normal household duties. Three weeks after Mary Verney had given birth to a son in 1647 she wrote to her husband noting that, although the baby was well, ‘for my self I am so very weak that ontell (until) yesterday, since I was brought to bed, I have neavor been able to sitt up ann hower at a time’. She was also ‘so tormented with pains’ in her head, that if she sat up for more than a quarter of an hour ‘it puts me into such sweates as I am not able to endure itt.’ She lamented that if only her headache was to abate, ‘I should recover my strenth apace’.^76^ In a similar episode, Alice Thornton recorded after the birth of her first child that she suffered from a ‘desperate & dangerous sickness’ where she was ‘brought soe weak that my speech was taken from me, not being able to call for any helpe’. She too was rendered immobile after birth, noting ‘for many weeks’ she was not able to turn her ‘weary bones in bed nor helpe my selfe in the least’.^77^ Thornton’s experience was one of complete loss of control; she was unable to walk or speak. At the time when prescriptive medical and religious texts stressed that women ought to be capable of being an active carer in their infant’s life, women who experienced elongated recovery periods were unable to care for themselves and required feeding, medicating and supervision.

One important factor which significantly complicated the ability to return to normal functioning was if a family member, particularly the newborn infant, was unwell. Olivia Weisser has recently described how early modern women ‘commonly looked to others as models of suffering and attributed their own illness and recovery to negative or positive affective relations’, in contrast to men who ‘tended to privilege their own bodily experiences over the words and opinions of others’.^78^ One reason for this was that women’s bodies were understood in a humoral sense to be wetter, and thus more impressionable. The passions of the soul had a greater bodily effect, something that was constructed through ‘models’ of suffering provided by the illness narratives of friends and family. During periods of poor health, emotional distress could significantly curtail the ability to recover, and for newly delivered women, during the month or months in which they struggled to excrete the fluid of the pregnancy, their bodies were additionally impressionable.^79^ In this way, recently delivered women were especially vulnerable to the bodily impacts of the emotions, brought about by grief or distress, something Weisser has termed ‘mimetic’ illness or suffering.

Isaac Archer recorded in 1679, as his wife began to show promising signs of recovery, that a sudden deterioration in the health of the newborn drove his wife back into grave, life threatening illness; ‘the fits of the child grew worse, which grieved my wife, and sett her back’.^80^ A similar tale is found in Alice Thornton’s meditations. Newly delivered and recuperating in bed, Alice relayed how her other daughter, Nally, a newly weaned toddler, ‘fell into a desperate fit of convulsions […] her breath stoping & grew blackish in her face’. Her condition continued to worsen. Midwives and Alice’s relatives took turns to watch the gravely ill infant. Alice recalled,

During this poore childes illness, I was almost at deaths doore my selfe by a great Ilness […] soe that my Aunt & friends did imagine I could not liue not durst they tell me how seake [sick] my sweete Nally was at that time least grief should haue despatched me hence. but they removed her in the cradle into the Parlour.^81^

Nally was shifted away to limit the impact of her illness on her mother’s health. The emotional toll of being confronted by the sounds and sights of a dying child, Alice and others believed, would stir up her humours so excessively, that she too would start to exhibit signs of near death like Nally.

When Lady Ann Fanshawe’s newborn died in 1645 she recorded ‘it cost me so dear that was ten weeks before I could go alone’. She was constantly monitored and unable to feed, clean or dress herself. Although Fanshawe had given birth in February it was not until May that she ‘went out of my chamber and to church’ to St John’s College, Oxford. Going to church was in a prescriptive model meant to signify recovery, however, Fanshawe still perceived herself to be suffering. She noted that although she was able to get to church and sit through the ceremony, afterwards she had to rest in the college gardens, ‘being very weak’.^82^

In addition to grief and distress, accidents could also set the progress of recovery back significantly. Two days after being churched, for example, Mary Smith had an ‘unlucky fall’ and injured her back. She was confined to ‘bed and chamber almost another monthe, I was so yell (ill) I feared some bone was amisse, but now I find it brused.’ In a letter to her brother she relayed that ‘this beinge the furst day I haue bein out of doors sense crismas’.^83^ Even when she regained enough strength to venture out of doors again, she stressed to her brother ‘I think I shall feele it agood while’ and apologised for her poor handwriting, a result of her afflictions. While returning to an internal estimation of a former self was the unequivocal aim of after birth care regimes in early modern England, feeling healthy did not always correspond to the prescriptive timetables.

Breast inflammation and scarring were particularly common problems after birth which often caused lasting scars and suffering for which there was little cure. Florence Smyth, for example, was ‘still much tormented w[it]h payne under her breast, & be very weake’ months after giving birth in 1637.^84^ Likewise, Hannah Woodford, wife of Robert, continued to suffer with sore breasts for three months after birth. Shortly after the delivery of the Woodford’s second child in 1637, Robert recorded in his diary ‘Litle John sucks well blessed be the Lord and is mending thanks be to god.’ His wife, however, was ‘troubld with sore & tender breasts’.^85^ Over these months Robert wrote almost daily in his diary about his wife’s breasts and prayed to God ‘heale them and make her a Joyfull mother’.^86^ Such ailments were taken seriously and curtailed normal life, despite the period of lying-in being over. Hannah was churched on 3 September 1637, but this ceremony had to take place at home rather than at church because she was too ill to travel.^87^ On 11 September Robert recorded that one ‘Nurse Woodnot’ was sent away.^88^ Nurse Woodnot was presumably contracted to care for Hannah, rather than the child, given Mrs Rushworth, a family friend and former servant, had been suckling the baby since 31 August.^89^ The nurse’s prolonged stay highlights the significant toll that breast ailments could have on women in returning to health. Hannah continued to ail. On 2 October Robert recorded that those attending his wife predicted that her breasts would split or ‘break’ with the inflammation. He did not specify whether these ‘others’ were midwives, family friends, unlicensed healers, physicians or a combination.^90^ Robert continued to comment on the condition of Hannah’s breasts throughout the year and on 10 December remarked that she was too unwell to attend church.^91^

Nehemiah Wallington’s wife similarly suffered from sore and inflamed breasts in the months after birth. He recorded:

About one month after my wife was brought abead shee begun to have sore brests so that the childe did not sucke for three days together, that wee ware faine to put it forth to a norse into the country for my wife was in such paine with her breasts that she coulde take no rest for many days and nights together and was in great paine for sixe weeks together: and then it pleased the Lord my God to blesse the meanes that we did use and healed her breastses his Name bee praised for he is a very mercifull and pitifull tender hearted Father.^92^

Robert Woodford and Nehemiah Wallington recorded their wives’ inflamed breasts in a way that suggested they saw these conditions as serious and dangerous. This was in direct contrast with the relative ambivalence with which medical authors considered childbirth complications that were not a result of insufficient or uncontrollable bleeding. Pechey, for example, instructed women to just leave swellings in the breast ‘if no other Symptom attend them’. This was explained simply as ‘the over eagerness of the milky Ferment’, and would easily disappear.^93^

Medical authors did not consider breast problems that occurred more than a month after childbirth life-threatening because the contents of breasts were understood to have been concocted and purified and because lochia had been excreted. It seems in some ways illogical that physicians did not equally seek to ‘rid’ women of their breast milk. Because this blood had been purified, and the lochia long gone, the inflammation was not attributed to insufficient purging. Furthermore, breast ailments tended not to occur during pregnancy or immediately after birth; they were perceived not as an immediate product of the presence of the infant and childbirth, but rather by a later weakness in the mother.

As with recovery more generally, infant illness and distress could intensify and worsen maternal health and breast ailments. Alice Thornton explained that her newborn’s fits caused her to produce less and poor quality breast milk, ‘This ill fitt hindered my milke much.’ Once her child repaired, she too was quick to recover: ‘I recruted fast & within a fortnight had gotten the milke again into my breasts & my deare babe Betty did sucke euery day of me & I was ouerioyed in the great blessing.’ The joy of breastfeeding however was short lived. Sitting ‘in my chaire & giueing my child sucke’ one evening, she heard ‘one of the maids creid out of the nursery that my childe Naly was either dead or dieing, which soe affrighted me being but weake that an Illnesse came in force upon me’. She had to be helped to her bed and although her milk eventually returned, her own mother was so distressed by her illness that ‘she would not lett me giue sucke, although I extreamely desired it, & att the months end I was forced to drie my breasts which grew full & had indangered to bring me ill againe’.^94^ Drying the breasts did not involve removing existing breast milk. Rather, as Culpeper instructed, women should place ‘repellers under the Arm-pits, as Unguent of Roses Cerot of Sanders, dissolved in vinegar, and to the breasts apply a Cataplasm of Bean’.^95^ The breasts could be ‘fomented’ with mint, dill, smallage and the bruised leaves placed to the breasts.^96^ This shows that in the months after childbirth, breast ailments were not necessarily attributed to internal corrupt matter, but instead to less troubling inflammation.

For some women their childbearing narratives did not seem to ever have a clear end. In a series of letters that Anne Dormer, an Oxfordshire gentlewoman, penned to her sister, Elizabeth Trumbull, between 1685 and 1691, she described her struggles with persistent poor health and an unhappy marriage. In one letter she wrote that she was thankful ‘I haue not *great* paines only a languishing kind of unaccountable illness which keepes me pale and leane’.^97^ She placed her ongoing illness into a bodily narrative which involved childbearing, ‘for I haue a body like a horss [horse] to tug through all I haue endured of illness and childing’.^98^ Her body was diminished: she had lost her appetite, had difficulty moving and could not sleep. Comparing herself to a horse served to emphasise the struggles she had faced—it was a wonder she had not died. These letters were written long after she had given birth and been purged, and suggest that the difficulties of recovering from childbirth and persistent poor health were to some degree attendant to a particular reproductive life-stage. In one letter she told Elizabeth ‘all French Wine is too rakeing for my carcase which grows still leaner’; ‘if I get but one good [night of sleep] me thinks I am so well I can complain of nothing’ and she had avowedly given up hope of ‘going out farther than my garden’.^99^

Dormer accepted her illness as a divine test that had to be borne patiently, a key tenet of early modern religiosity. Her resignation to the permanence of her afflictions was notable even in these terms. Her only ‘cordiall’, or relief, and ‘hope in reviving in the world’ was to be found in time spent with her friends, children and consuming a ‘dish of chocolate’.^100^ Anne found comfort amidst her troubles, but perceived her body to be permanently disordered as a result of illness and ‘childing’. Despite the optimism of seventeenth-century medical texts that bleeding could make a woman well, there were some ills that could not be rectified by this regimen. Anne may have been perceived by the practitioners she saw as being cleansed and divested of corrupting matter, but she felt the continuing impact of childbirth. Perhaps Anne was weak and diminished because she had bled excessively and for too long. Perhaps her pregnancy and labour caused debilitating tears, incontinence or injuries. Whatever the explanation, Anne was not recovered, but in a sense her ‘childing’ narrative continued. Recovery in this way was measured by when a woman felt she had returned to normal functioning. Dormer’s account suggests that in childbearing and illness narratives, this might never be within reach.

## Conclusions

In prescriptive medical and religious models of childbirth a new mother was ‘well’ when she had been delivered of a child and a placenta, and had bled away the lochia. This was meant to occur in the month after delivery. Historians have uncritically conflated the cessation of bleeding, churching and returning to health as being one and the same thing. As I have shown, these were three separate issues and being technically healthy and feeling healthy were very different things. Some women like Florence Smyth recovered precociously quickly, while others like Dormer continued to feel their bodies were affected by childbirth. One imagines for women not in a financially and socially privileged enough position to enjoy a month lying-in, the rituals of churching and leaving the house, bore even less relationship to whether they felt well.

Men *and* women constructed narratives about childbearing in their life-writing. These stories branched different textual forms and were embedded in a performative process in which reproduction was knitted into autobiographical reflections that sometimes consciously sought to publicly validate an individual and their behaviour as religiously exemplary. These narratives did not simply end when a child was delivered, but when a new mother felt she had returned to her former self. In this way, I have shown how having babies was seen as an ongoing, fluid process in which women continued to ‘labour’. This process could be curtailed and complicated by emotional distress brought about by the death or illness of a family member, or by accidents.

So much of the scholarship on early modern childbirth has been concerned with whether or not the birthing chamber was a supportive space, but only Ulinka Rublack has called into question its wholly feminine nature.^101^ This is because by focusing on the rituals surrounding the actual delivery, in which men would not have been physically present, historians have missed their significance in shaping childbearing. Male relatives wrote letters to each other detailing pregnancy symptoms, deliveries and the progress of lochial bleeding candidly. Similarly, men like Isaac Archer, Robert Woodford and Nehemiah Wallington wrote almost daily about their wives’ recoveries. This strikes a chord with Magdalena S. Sánchez’s recent findings in the Court of Turin, 1585–1597, that childbirth was not necessarily seen as a ‘strictly feminine event’ by Catalina Micaela and her husband Carlo Emanuele, Duke of Savoy.^102^ My findings about male involvement and interest in maternal health similarly support the arguments of Elaine Leong and Lisa Wynne Smith that early modern English domestic medicine was not just a female domain.^103^ Likewise, Sara Read and Jennifer Evans’ examination of miscarriage, have shown that men acted as ‘commentators and informers, as seekers of medical care and as repositories of medical knowledge’.^104^

Recovery from childbirth was not a clear, formulaic process as medical literature would imply. Women might have bled away the remnants of pregnancy and in this internal sense have been ‘well’ but continued to look and feel ill long after they had given birth. They did not do this in isolation, however, but in concert with family members, male and female.

## Footnotes

1 Dr Denton to Ralph Verney, 4 July 1647, Frances Parthenope Verney, ed., *Memoirs of the Verney Family During the Civil War* (1892), 272.

2 Adrian Wilson, ‘The Ceremony of Childbirth and its Interpretation’, in Valerie Fildes, ed., *Women as Mothers in Pre-Industrial England* (London: Routledge, 1990); *Ritual and Conflict: The Social Relations of Childbirth in Early Modern England* (Farnham: Ashgate, 2013); Patricia Crawford, ‘The Construction and Experience of Maternity in Seventeenth-Century England’, in *Women as Mothers*; David Cressy, *Birth, Marriage, and Death: Ritual, Religion, and the Life-Cycle in Tudor and Stuart England* (Oxford: Oxford University Press, 1999). Other studies have troubled this assumption that women bonded and supported each other in childbirth: Linda A. Pollock, ‘Childbearing and Female Bonding in Early Modern England’, *Social History*, 1997, 22, 286–306 and Laura Gowing, *Common Bodies: Women, Touch and Power in Seventeenth-Century England* (New Haven, London: Yale University Press, 2003). Ulinka Rublack’s has, in contrast, stressed male involvement in preparation for labour and the period after birth in, ‘Pregnancy, Childbirth and the Female Body in Early Modern Germany’, *Past & Present*, 1996, 150, 84–110.

3 Gowing, *Common Bodies*, 150.

4 Sara Read, *Menstruation and the Female Body in Early Modern England* (Basingstoke: Palgrave Macmillan, 2013), 164.

5 Keith Thomas, *Religion and the Decline of Magic* (Harmondsworth: Penguin, 1978, 1991 reprinted), 43.

6 Crawford, ‘The Construction and Experience’, 25 and ‘Attitudes to Menstruation in Seventeenth-Century England’, *Past & Present*, 1981, 91, 47–73.

7 David Cressy, ‘Purification, Thanksgiving and the Churching of Women in Post-Reformation England’, *Past & Present*, 1993, 141, 106–46.

8 Cressy, ‘Purification, Thanksgiving and the Churching of Women’, 144. Wilson makes a similar argument in ‘The Ceremony of Childbirth’.

9 Cressy, ‘Purification, Thanksgiving and the Churching of Women’, 145.

10 Gail Kern Paster, *Body Embarrassed: Drama and the Disciplines of Shame in Early Modern England* (New York: Cornell University Press, 1993), 195.

11
*Ibid*., 197.

12 James Daybell, *The Material Letter in Early Modern England: Manuscript Letters and the Culture and Practices of Letter-Writing, 1512–1635* (Basingstoke, New York: Palgrave Macmillan, 2012), 10. Effie Botonaki has commented that ‘The emergence, form, and proliferation of diary writing in the sixteenth and seventeenth centuries owe a great deal to the popular practice of self-examination advocated by the Protestant faith’, in ‘Seventeenth-Century Englishwomen’s Spiritual Diaries: Self-Examination, Covenanting, and Account Keeping’, *Sixteenth Century Journal*, 1999, 30, 3–12, 3.

13 Lucinda McCray Beier, *Sufferers and Healers: The Experience of Illness in Seventeenth-Century England* (London: Routledge & Kegan Paul, 1987); Amanda E. Herbert, ‘Gender and the Spa: Space, Sociability and Self at British Health Spas, 1640–1714’, *Journal of Social History*, 2009, 43, 361–83; Pollock, ‘Childbearing and Female Bonding’; Linda A. Pollock, ‘Anger and the Negotiation of Relationships in Early Modern England’, *Historical Journal*, 2004, 47, 567–90; Lisa Wynne Smith, ‘“An Account of an Unaccountable Distemper”: The Experience of Pain in Early Eighteenth-Century England and France’, *Eighteenth-Century Studies*, 2008, 41, 459–80; ‘The Relative Duties of a Man: Domestic Medicine in England and France, ca. 1685–1740’, *Journal of Family History*, 2006, 31, 237–56; ‘The Body Embarrassed? Rethinking the Leaky Male Body in Eighteenth-Century England and France’, *Gender & History*, 2011, 23, 26–46; Olivia Weisser, ‘Boils, Pushes, and Wheals: Reading Bumps on the Body in Early Modern England’, *Social History of Medicine*, 2009, 22, 321–39; Olivia Weisser, ‘Grieved and Disordered: Gender and Emotion in Early Modern Patient Narratives’, *Journal of Medieval and Early Modern Studies*, 2013, 43, 247–74.

14 Naomi Tadmor, *Family and Friends in Eighteenth-Century England: Household, Kinship and Patronage* (Cambridge: Cambridge University Press, 2001), 19.

15 Adam Smyth, *Autobiography in Early Modern England* (Cambridge, 2010), 2.

16 Margaret Ezell, *Writing Women’s Literary History* (Baltimore: Johns Hopkins University Press, 1993), 35. See Alice Thornton, British Library (henceforth BL) Add MS 88897/1 and 2.

17 Gary Schneider, ‘Affecting Correspondence: Body, Behaviour, and the Textualization of Emotion in Early Modern English Letters’, *Prose Studies*, 2000, 23, 31–62. See also, Fay Bound, ‘“Writing the self?”: Love and the Letter in England, c. 1660-c. 1760’, *Literature and History*, 2002, 11, 1–19.

18 Mary E. Fissell, ‘The Marketplace of Print’, in Mark S. R. Jenner and Patrick Wallis, eds, *Medicine and the Market in England and its Colonies, c. 1450-c.1850* (Basingstoke: Palgrave Macmillan, 2007), 114.

19 Hannah Newton, ‘“Nature Concocts & Expels”: The Agents and Processes of Recovery from Disease in Early Modern England’, *Social History of Medicine*, 2015, 28, 465–86, 466.

20 Alice Thornton, BL Add MS 88897/1, 155–6.

21 Nicholas Culpeper, *Culpeper’s Directory for Midwives: or, A guide for women* (London, 1662), 186.

22 John Pechey, *A General Treatise of the Diseases of Maids, Bigbellied Women, Child-bed Women, and Widows Together with the Best Methods of Preventing or Curing the Same* (London, 1696),155.

23 Jane Sharp, in Elaine Hobby, ed., *Midwives Book, Or the Whole Art of Midwifry Discovered* (New York, Oxford: Oxford University Press, 1999), 167.

24 Pechey, *A General Treatise of the Diseases of Maids*, 155–6.

25
*Ibid*., 155.

26 Culpeper, Chapter 7 ‘Of Watching, Doting and Epilepsie of Women in Childbirth’, 194–5.

27
*Ibid*., 195–6.

28 Sharp, *The Midwives Book*, 180.

29 Culpeper, *Culpeper’s Directory for Midwives*, 194.

30 Cathy McClive, *Menstruation and Procreation in Early Modern France* (Farnham, 2015), 144. See also, Gianna Pomata, ‘Menstruating Men: Similarity and Difference of the Sexes in Early Modern Medicine’ in Valerie Finucci and Kevin Brownlee, eds, *Generation and Degeneration: Tropes of Reproduction in Literature and History from Antiquity through Early Modern Europe* (Durham, 2001), 109–52.

31 Sharp, *The Midwives Book*, 168.

32
*Ibid*.

33
*Ibid*., 84.

34 Sara Read argues that the terminology of postpartum bleeding standardised in the seventeenth century in Read, *Menstruation and the Female Body*, 147.

35 Pechey, *A General Treatise of the Diseases of Maids*, 161.

36
*Ibid*., 156.

37
*Ibid*., 165–6.

38 Pomata, ‘Menstruating Men’, 137–8; Wendy D. Churchill, *Female Patients in Early Modern Britain: Gender, Diagnosis, and Treatment* (Farnham: Ashgate, 2012), 93–123. See also: McClive, *Menstruation and Procreation*; Read, *Menstruation and the Female Body*; Jennifer Evans, ‘“Gentle Purges corrected with hot Spices, whether they work or not, do vehemently provoke Venery”: Menstrual Provocation and Procreation in Early Modern England’, *Social History of Medicine*, 2012, 25, 2–19 and Crawford, ‘Attitudes to Menstruation’.

39 Pechey, *A General Treatise of the Diseases of Maids*, 158.

40 Sharp, *Midwives Book*, 179.

41 See, Valerie Fildes, *Breasts, Bottles, and Babies: A History of Infant Feeding* (Edinburgh: Edinburgh University Press, 1986); Victoria Sparey, ‘Identity-Formation and the Breastfeeding Mother in Renaissance Generative Discourses and Shakespeare’s *Coriolanus’*, *Social History of Medicine*, 2012, 25, 777–94.

42 Culpeper, *Directory for Midwives*, 67.

43 Sharp, *The Midwives Book*, 175.

44 Charles Cheyne to Charles Cavendish, Viscount Mansfield, 18 May 1656, Portland Papers, Nottingham University Library, PW1/84.

45 Charles Hatton to Christopher Hatton, 1678, BL, add MS 29571, f. 472.

46 Thomas Smyth to Edward Phelips, October 1641, Somerset Record Office (henceforth SRO), DD/PH/224133.

47 Francis Thornhough to Oliver St John, 13 October 1674, Bedfordshire and Luton Archives and Records Service, J1469.

48 Thomas Smyth to Edward Phelips, October 1641, SRO, DD/PH/224133.

49 See, Jennifer Evans, *Aphrodisiacs, Fertility and Medicine in Early Modern England* (Woodbridge, 2014).

50 Thomas Smithe to Thomas Smyth, 28 March 1637, Bristol Record Office (BRO), AC/c54/5.

51 Laura Gowing, ‘Secret Births and Infanticide in Seventeenth-Century England’, *Past & Present*, 1997, 156, 87–115, 99.

52
*Ibid*., 99.

53 Thomas Bentley, *The Monument of Matrones Conteining Seuen Seuerall Lamps of Virginitie, or Distinct Treatises* (London, 1582). See, Colin B. Atkinson and William P. Stoneman, ‘“These griping greefes and pinching pangs”: Attitudes to Childbirth in Thomas Bentley’s *The Monument of Matrones* (1582)’, *Sixteenth Century Journal*, 1990, 21, 193–203. Sharon Howard has argued that Alice Thornton similarly drew on ‘discourses of martyrdom’ in describing the pain of childbirth in ‘Imagining the Pain and Peril of Seventeenth-century Childbirth: Travail and Deliverance in the Making of an Early Modern World’, *Social History of Medicine*, 2003, 16, 367–82.

54 Isaac Archer, 13 June 1672, in Matthew Storey, ed., *Two East Anglian Diaries, 1641–1729: Isaac Archer & William Coe* (Woodbridge: Boydell Press, 1994), 154.

55
*Ibid*., 154.

56 For a discussion of casebooks see Lauren Kassell, ‘Casebooks in Early Modern England: Medicine, Astrology, and Written Records’, *Bulletin of the History of Medicine*, 2014, 88, 403–33.

57 Joan Lane, ‘Hall, John (1574/5?–1635)’, *Oxford Dictionary of National Biography*, Oxford University Press, 2004 online edn, Jan 2008 <http://www.oxforddnb.com/view/article/11968?docPos=3>, accessed 25 November 2016.

58 Olivia Weisser, *Ill Composed: Sickness, Gender, and Belief in Early Modern England*, New Haven: Yale University Press, 2015.

59 John Hall, *c*. 1650, Observation (henceforth obs.) 154, in Joan Lane, ed., *John Hall and his Patients: The Medical Practice of Shakespeare’s son-in-law* (Stratford-upon-Avon, 1996), *c*. 1650, 282–5. Hall did not visit her but treated her based on reports he had heard.

60
*Ibid*., *c*. 1634–5, obs. 156, 288–91.

61
*Ibid*., *c*. 1616, obs. 28, 50–1.

62 Archer, 1 August 1676, *Two East Anglian Diaries*, 189.

63 Hall, *c*. 1617, obs. 59, *John Hall*, 109.

64
*Ibid*., *c*. 1620, obs. 65, 118–19.

65
*Ibid*., *c*. 1642, obs. 37, 70.

66 Thornton, BL Add MS 88897/1, 155–6.

67 Sharp, *The Midwives Book*, 150.

68 Culpeper, *Directory for Midwives* (1676), 193. The first recorded instance of an episiotomy was in 1742, and thus I am assuming that tears were accidental rather than conscious incisions. See, H. G. Dahlen, C. S. E. Homer, N. Leap and S. Tracy, ‘From Social to Surgical: Historical Perspectives on Perineal Care during Labour and Birth’, *Women and Birth*, 2011, 24, 105–11.

69 Culpeper, *Directory for Midwives*, 194.

70 It is unclear whether ‘baths’ in this text referred to submerging the body in water, or was achieved with clouts.

71 John Pechey, *The Compleat Midwife’s Practice Enlarged in the most weighty and high concernments of the Birth of Man* (1698), 122.

72
*Ibid*., 125.

73 James Yonge, 7 January 1681, in F. N. L. Poynter, ed., *The Journal of James Yonge [1647–1721]: Plymouth Surgeon* (London: Longmans, 1963), 147.

74 Charles Trelawny to Sidney Godolphin, 7 November 1700, BL Add MS 28052, f. 100.

75 Elizabeth Smyth to Thomas Smyth, *c.* 1629, BRO, AC/C/48/12.

76 Mary Verney to Ralph Verney, 24 June 1647, *Memoirs of the Verney Family*, 267–8.

77 Thornton, BL, Add MS 88897/1, 133.

78 Weisser, *Ill Composed*, 3. See also, ‘Grieved and Disordered’.

79 Infants were also undergoing a process of excretion during this period after birth. For a more detailed account of this and the care of infants, see my forthcoming chapter ‘“Ordering the Infant”: Caring for Newborns in Seventeenth-Century England’, in Sandra Cavallo and Tessa Storey. eds, *Conserving Health in Early Modern Culture. Bodies and Environments in Italy and England* (In press, Manchester, Manchester University Press).

80 Archer, 1 August 1679, *Two East Anglian Diaries*, 189.

81 Thornton, BL Add MS 88897/1, 138.

82 John Loftis, ed., *The Memoirs of Anne, Lady Halkett and Ann, Lady Fanshawe* (Oxford: Clarendon Press, 1979), 31–2.

83 Mary Smith to Thomas Smith, 30 March *c*. 1630, BRO, AC/C53/6. This could have been the daughter that she gave birth to on 8 August 1633.

84 Thomas Smith to Thomas Smyth, 28 March 1637, BRO, AC/C 55/2.

85 Woodford, 1 September 1637, *Diary*, 104.

86
*Ibid*., 104.

87
*Ibid*., 3 September 1637, 107.

88
*Ibid*., 11 September 1637, 111.

89
*Ibid*., 31 August 1637, 104; 1 September 1637, 104.

90
*Ibid*., 2 October 1637, 120.

91
*Ibid*., 10 December 1637, 150.

92 Nehemiah Wallington, December 1624, in David Booy, ed., *The Notebooks of Nehemiah Wallington, 1618–1654: A Selection* (Aldershot: Ashgate, 2007), 412.

93 Pechey, *A General Treatises of the Diseases of Maids*, 175.

94 Thornton, BL Add MS 88897/1, 138–9. Alice Thornton asked someone known to her to breastfeed her infant, rather than an unknown country nurse. Dafeny Lightfoote, who was a family friend and occasional servant of the Thorntons, breastfed the infant until she herself became pregnant.

95 Culpeper, *Directory for Midwives*, 187.

96
*Ibid*., 187.

97 Anne Dormer to Elizabeth Trumbull, 9 August *c.* 1690, BL, Add MS 72516, f. 157. My emphasis.

98
*Ibid*.

99 Dormer to Trumbull, 10 September *c.* 1690, BL, Add MS 72516, f. 167.

100
*Ibid*.

101 Rublack, ‘Pregnancy, Childbirth and the Female Body’.

102 Magdalena S. Sánchez, ‘“I would not feel the pain if I were with you”: Catalina Micaela and the Cycle of Pregnancy at the Court of Turin, 1585–1597’, *Social History of Medicine*, 2015, 28, 445–64. Advanced access published online 5 March 2015.

103 Elaine Leong, ‘Making Medicines in the Early Modern Household’, *Bulletin of the History of Medicine*, 2008, 82, 145–68; ‘Collecting Knowledge for the Family: Recipes, Gender and Practical Knowledge in the Early Modern English Family’, *Centaurus*, 2013, 55, 81–103; Lisa Wynne Smith, ‘The Relative Duties of a Man’.

104 Sara Read and Jennifer Evans, ‘“Before midnight she had miscarried“: Women, Men, and Miscarriage in Early Modern England’, *Journal of Family History*, 2015, 40, 3–23, 17.

